# Contributions of Colloidal Forces to the Heterogeneous Separation of Stable Oil-In-Water Emulsions

**DOI:** 10.1021/acs.langmuir.4c03056

**Published:** 2024-10-28

**Authors:** Roi Bar-On, Ofer Manor

**Affiliations:** † Applied mathematics department, Technion - Israel Institute of Technology, Haifa, 3200000, Israel. Currently at Institut de Biologie de l’École Normale Supérieure ENS, Paris 75005, France; ‡ Department of Chemical Engineering, Technion - Israel Institute of Technology, Haifa 3200000, Israel

## Abstract

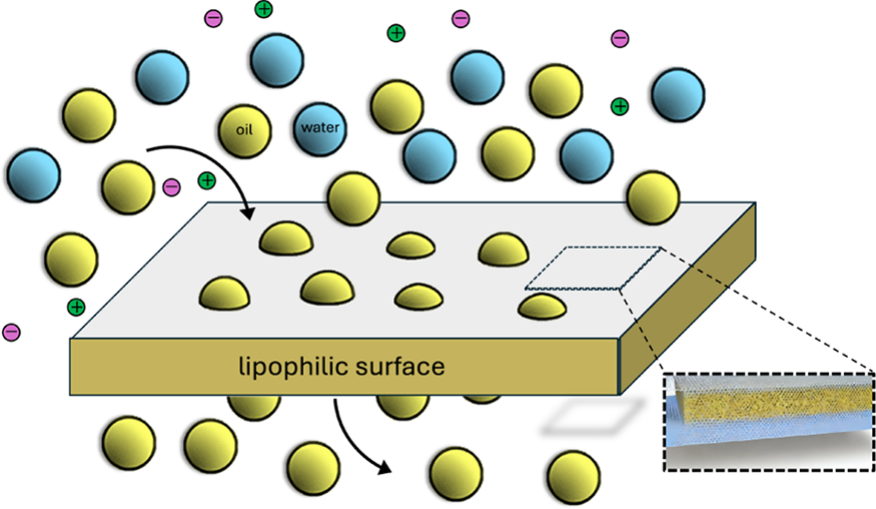

We use theory to study the distribution of spherical emulsion oil droplets in water near a lipophilic surface as a guideline for designing membranes for oil/water phase separation. Heterogeneous phase separations are shown in our laboratory using hydrophilic and hydrophobic membrane designs, where the affinity of the membrane surface to one of the phases in the mixture locally increases its concentration. Considering a colloidal emulsion (nano- to microemulsions) of spherical and noncoalescing droplets, we assess the contribution of colloidal forces, i.e., van der Waals, electrical double layer, and hydrophobic interactions and the finite size of the droplets to the accumulation of spherical emulsion droplets near a surface. We use our theory to study an experiment-inspired case study and find that an isolated lipophilic membrane surface in contact with an oil-in-water emulsion supports the oil-enriched emulsion phase in a thin layer near the membrane surface, suggesting that a membrane pore size comparable to this thickness should support oil-enriched emulsion in the membrane pores and hence past the membrane.

## Introduction

**Figure 1:**
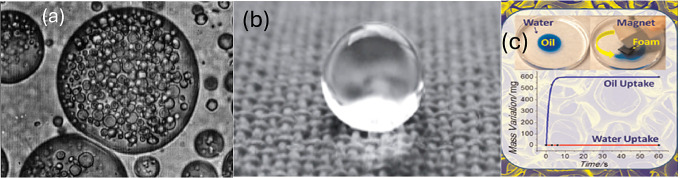
(a) oil and water droplets as part of emulsions stabilized solely by colloidal particles.^4^ Reprinted from ELSEVIER, 113, R. Wahi, L. A. Chuah, T. S. Y. Choong, Z. Ngaini, M. M. Nourouzi, oil removal from aqueous state by natural fibrous sorbent: an overview, Separation and Purification Technology, 51–63, Copyright (2013), with permission from Elsevier. (b) Polyester materials with superwetting silicone nanofilaments for oil–water separation and selective oil absorption.^12^ Reprinted from Advanced Functional Materials, 21, J. Zhang, S. Seege, Polyester materials with superwetting silicone nanofilaments for oil–water separation and selective oil absorption, 4699–4704, Copyright (2011), with permission from John Wiley and Sons. (c) Magnetically driven floating foams for the removal of oil contaminants from water.^8^ Reprinted (adapted) with permission from P. Calcagnile, D. Fragouli, I. S. Bayer, G. C. Anyfantis, L. Martiradonna, P. D. Cozzoli, R. Cingolani, A. Athanassiou, Magnetically driven floating foams for the removal of oil contaminants from water, ACS nano 6 (6) (2012) 5413–5419. Copyright 2012 American Chemical Society.

Oil-polluted water is produced in large quantities in both domestic and industry utilities, such as mining, textiles, foods, petrochemicals, metal/steel industries, and biofouling.^1^ This has become an extremely common pollution source and is considered to be a serious global environmental concern,^2,3^ which necessitates new strategies for domestic and small industry scale separation of oil from water. In recent years, a rapid growth can be observed in research into separation methods of oil-in-water emulsions and other chemicals.^4^ Oil skimmers or booms^5^ have been used to purify oil-in-water and water-in-oil mixtures in industry, and porous materials, such as sponges,^6,7^ foams,^8,9^ and textiles^9^,^10−12^ have been commonly used to absorb oils to address oil spillages, see Figure 1. The latter approach introduces many times a secondary pollution of the environment and land contamination since recycling/reuse of these materials and the adsorbed oil is typically difficult and time-consuming; hence, they are generally burned or buried in the ground. Here, we use theory to study oil-in-water phase separation using porous membranes.^12^,^13−19^

It was shown that membranes of specific affinity to water or oil allow for the passage of the favorable phase through the membrane pores, while repealing the opposite phase.^13,19^ The substrates of membranes are hydrophobic or hydrophilic. For example, an hydrophobic poly vinylidene fluoride (PVDF) membrane was used to separate various water-in-oil emulsions including surfactant-free and surfactant-stabilized emulsions with droplet sizes from the micro to the nanometer range.^14^ Driven by gravity, the membrane appeared to exhibit a good oil–water separation efficiency. Moreover, a hydrophilic hydrogel polyacrylamide was coated with a mesh consisting of rough nanostructured hydrogel coatings and microscale porous metal substrates to separate various oil-in-water mixtures.^16^ In addition, Zhang et al.^13^ have presented membranes of variable oil wettability based on polyurethane sponges, and Wen et al.^17^ presented a zeolite-coated mesh film for oil–water separation. Using theory, Xie et al.^20^ explored the system of crude oil–brine-rock in the molecular level to pinpoint the mechanisms of wettability alternation to maximize oil recovery. Han et al.^21^ offered a mathematical model to analyze the water film thinning and the adherence of oil droplets on a surface in a laminar flow field. Sanaei et al.^22^ presented a surface complexation model to calculate the zeta-potential at oil and rock surfaces while considering a water film between oil and rock using the DLVO theory (electrostatic and van der Waals (vdW) interactions between colloids). In addition, Guo and Kovscek^23^ modeled the influence of brine chemistry on the behavior of short-range non-DLVO forces, e.g., hydration and discrete ion charge effects at calcite surfaces to determine how wetting films are affected by the presence of salts. Moreover, Takeya et al.^24^ proposed a triple-layer surface complexation model to describe calcite–brine and kaolinite–brine interfaces. Their model was verified with zeta potential experiments at various pH and calcium, magnesium, and sulfate concentrations.

Here, we import ideas from the statistical physics of interactions between small molecules and atoms via their finite volume and van der Waals (vdW) and Coulombic interactions to predict the distribution of colloidal emulsion droplets next to a solid surface. We account for the finite volume of the emulsion droplets and for colloidal forces in the emulsion, e.g., hydrophobic, vdW, and electrical double layer (EDL) interactions using mean field strategies;^25^,^26,27^ some of these strategies show qualitative agreement with experiments, and others are employed to realize the dynamics of colloidal deposition from volatile liquids.^28,29^

The translation of statistical ideas that are usually developed for studying molecular systems to colloidal systems is not trivial. In the case of small molecules, the intermolecular interaction potential is usually comparable to the characteristic thermal energy *k*_B_*T*, where *k*_B_ and *T* are the Boltzmann constant and temperature, respectively. However, a straightforward description of colloidal particles using the same approach may be misleading. Colloidal particles (such as oil droplets), which range in size from 1 nm to approximately 10 μm, may undergo enthalpic interaction energies of many magnitudes of *k*_B_*T*s; the colloidal interactions are the integration of interaction between large numbers of molecular elements in the colloidal particles.^30^,^31−33^ In the case of vdW interactions, this difficulty was circumvented by Hamaker who calculated vdW interactions between colloidal particles by integrating vdW interactions between the molecular elements therein; the modern Lifshitz theory of vdW interactions between colloidal particles employs similar ideas by using macroscopic electromagnetic properties of the interacting masses, implicitly integrating electromagnetic contributions from their molecular building blocks. Using similar principles and borrowing ideas from the theory for the statistical physics of polymers,^34,35^ we treat colloidal particles as bunches of molecular elements; the total interaction potential between colloidal particles accounts for the integral contributions between the molecular segments therein, whereby the internal degrees of freedom of molecules in the particles are assumed to be a leading-order constant during the interactions. The approach is found to give a physical order of magnitude to the coefficients of a corresponding Ginzburg–Landau type energy functional, which are comparable to experimental findings.^36^

In the Theory and Results section, we give a mathematical model to describe the distribution of colloidal droplets next to a surface of a substrate and discuss its implications, and in the Summary and conclusions section, we conclude our findings.

## Theory and Results

We model emulsions of small oil droplets in water that are stabilized by sufficient surfactant coverage to prevent droplet coalescence with each other or with the oleophilic membrane at the time of heterogeneous phase separation. We further assume that the oil droplets are sufficiently small to be considered spherical and kinetically stable, so that they predominantly experience surface forces, e.g., hydrophobic, vdW, and EDL (colloidal) interactions; both requirements are satisfied for colloidal emulsion droplets.

### Colloidal Interactions

**Figure 2:**
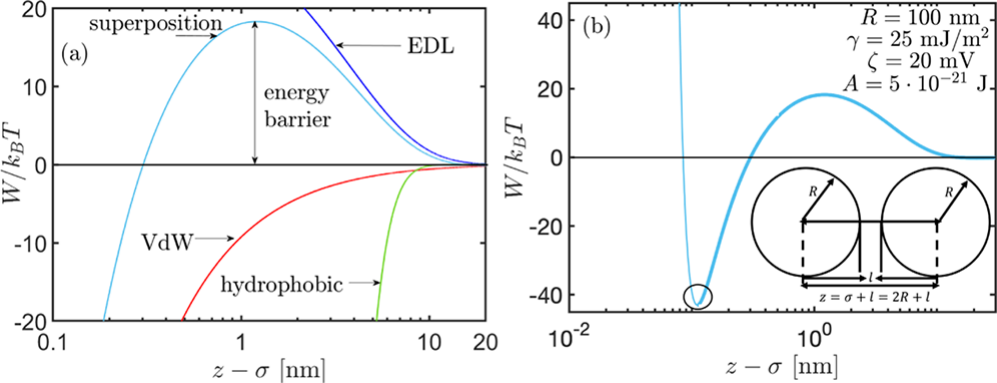
(a) vdW, EDL, hydrophobic, and their superposition interaction energies of 0.1 μm spherical colloidal particles. (b) Superposition of DLVO interaction energies and vdW repulsive correction interaction potential for 0.1 μm spherical colloidal particles, where the circles mark the minimum energy of interaction. Inset: illustration of two spherical colloidal oil droplets of radius *R,* which are separated by a center-to-center distance of *z* and a separation distance of *l*.

We assume that the oil droplets interact via vdW (*W*_vdW_), electrical double layer (*W*_edl_), and hydrophobic (*W*_hyd_) interaction potentials. Moreover, to account for the finite volume of colloidal drops, we assume that they remain spherical and invoke the classical hard sphere assumption in terms of the interaction potential, *W*_hs_. The total interaction potential between droplets is assumed as a superposition of the different interaction potentials given above and is given by
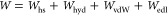
1We illustrate in this in Figure 2a.

The simplest model for the hard sphere repulsion is given by  at  and  at ,^37^ where *z* is the distance between the centers of two interacting spherical droplets of diameter σ, each (see the inset in Figure 2b). The hard sphere assumption does not fully capture the behavior of oil droplets, which are inherently deformable due to their fluid nature. However, droplets in the colloidal size regime that undergo thermal motion could be approximated to a leading order as spheres, which simplifies our analysis.

We approximate the vdW interaction potential between spheres using the classic geometrical expression^32,38^
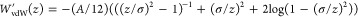
where *A* is the Hamaker constant, which is given a leading order by the Lifshitz theory as 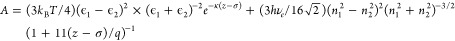
wherein ϵ_1_ and ϵ_2_ are the dielectric constants of oil and water, respectively, *h* is the Planck’s constant, ν_e_ is the electronic absorption peak frequency of water, *n*_1_ and *n*_2_ are the refractive indices of oil and water, and *q* ≈ 100 nm is the vdW characteristic retardation length due to the finite velocity of light. The pre-exponential term in the first component of the Hamaker constant yields the zero-frequency energy of the vdW interaction; the exponent accounts for the electrostatic screening of the zero-frequency field by the electrolyte solution;^32^ here,  is the Debye length, where *e* is the elementary charge, *z*_*i*_ is the *i*^th^ ion valence,  is the *i*^th^ ion bulk density, and ϵ_0_ is the permittivity of vacuum. The second term in the expression for the Hamaker constant accounts for the dispersion energy. We further account for a correction to the vdW interaction potential of opposite sign at small separations,
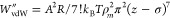
where ρ_m_ is the bulk solvent density, taken here is water. The correction appears directly from the analysis of vdW interactions between particles and is given in the Supporting Information. The leading-order vdW attractive and second order repulsive contributions to the particle–particle interaction potential, , are the main contributions to the interaction potential minimum at small particle–particle separation (Figure 2b), which in our analysis represents the equilibrium separation of attached but not coalesced droplets.^36^ To estimate the vdW interactions between drops, we calculated the Hamaker constant using characteristic hydrocarbon parameters. In particular, the dielectric constants of water and oil are assumed to be  at ambient temperature (298 K), The respective refractive indices are assumed to be , and the electromagnetic adsorption of water is assumed at .^32^

To approximate EDL interactions between particles, we assume the limit of small electrical potential; here the dimensionless EDL interaction potential is given by,^32^
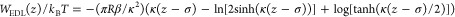
where  and α is the ionic strength in water. As a case study, to estimate the EDL interaction between drops, we consider the chemistry of the water,^39^ i.e., the content of the electrolyte solution, reported in the region where some of the previous heterogeneous phase separation experiments were conducted.^19^ As for the measurement, we assume the water phase contains calcium, , magnesium, , sodium, , chloride, , nitrate, , and sulfate,  at concentrations of  M,  M,  M,  M,  M, and  M, respectively, and additional unidentified ions y^–^ at a concentration of  M required to render the electrolyte solution electroneutral. The corresponding Debye length is 2.78 nm. Moreover, the zeta potential on the oil drops is extracted from data on emulsions of silicon oil-in-water^40^ and is approximately . We further estimate the hydrophobic interaction between two droplets using the approximation^41^
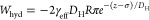
where *D*_H_ is a characteristic decay length of 1 nm and γ_eff_ is the effective oil–water interfacial tension (typically 50 mJ/m^2^).

**Figure 3:**
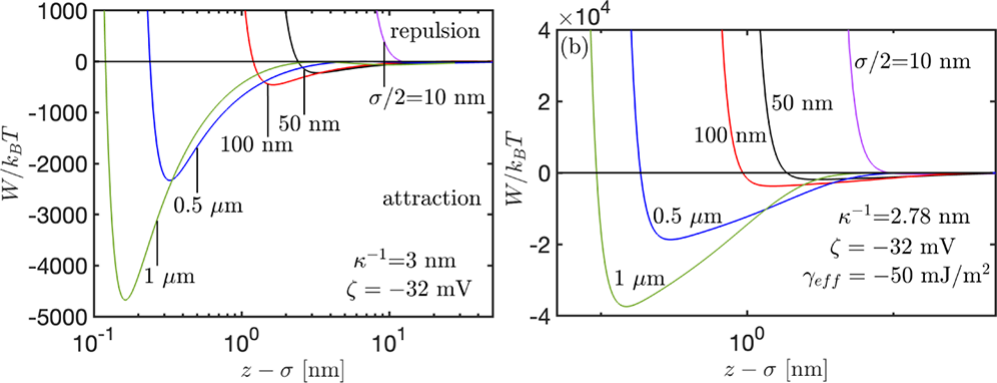
Droplet–droplet separation variation of the interaction potential for different droplet diameters, σ (a) with and (b) without hydrophobic interactions.

In Figure 3a, we plot the interaction potential between two oil droplets for the droplet diameter range,  μm. Moreover, in Figure 3b, we consider the contribution of hydrophobic forces between oil droplets, which dramatically increases the magnitude of the negative minimum energy. The interaction energies shown in the figures support energy minima, which increase in magnitude (the minima becomes deeper) with increasing the size of droplets. The minima in the interaction potential translates to the strength of attraction (adhesion) between droplets at the corresponding local equilibrium.

### Droplet Distribution

To model this problem, we represent the grand canonical ensemble of the emulsion in the absence of external forcing using^25,42^ in terms of the local drop density (), where **r** and μ are a spatial coordinate and the chemical potential, respectively, and  is the intrinsic Helmholtz energy, wherein *F*_id_ accounts for ideal contributions to the Helmholtz energy and the subsequent integral accounts for nonideal interactions between the emulsion drops. Assuming a slowly varying density, we expand  in terms of ρ to obtain , where  accounts for local contributions to  and  accounts for the leading-order nonlocal contributions; the linear expansion term vanishes over the volume integral, since it is an odd function of **r**. The result is the classical square gradient approximation (SGA) of the energy functional, which dates back to vdW^43,44^ and is well known following the work of Landau in the context of phase transitions^45^ and of Cahn and Hilliard in the context of interfaces.^46^

We further represent the emulsion using the nonideal gas equation , where *p* and  are pressure and the virial representation of deviations from ideal gas, respectively, wherein  is the second virial coefficient, which accounts for interactions between two particles at a time. The intrinsic Helmholtz energy, relatively to the emulsion bulk density ρ_0_, is represented by the expression
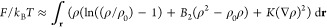
where the first term of the integral is the ideal gas contribution to Helmholtz energy, *F*, the second term is the local nonideal gas contribution, embedded in the second virial coefficient *B*_2_, and the last term accounts for the leading-order non local contributions.

**Figure 4:**
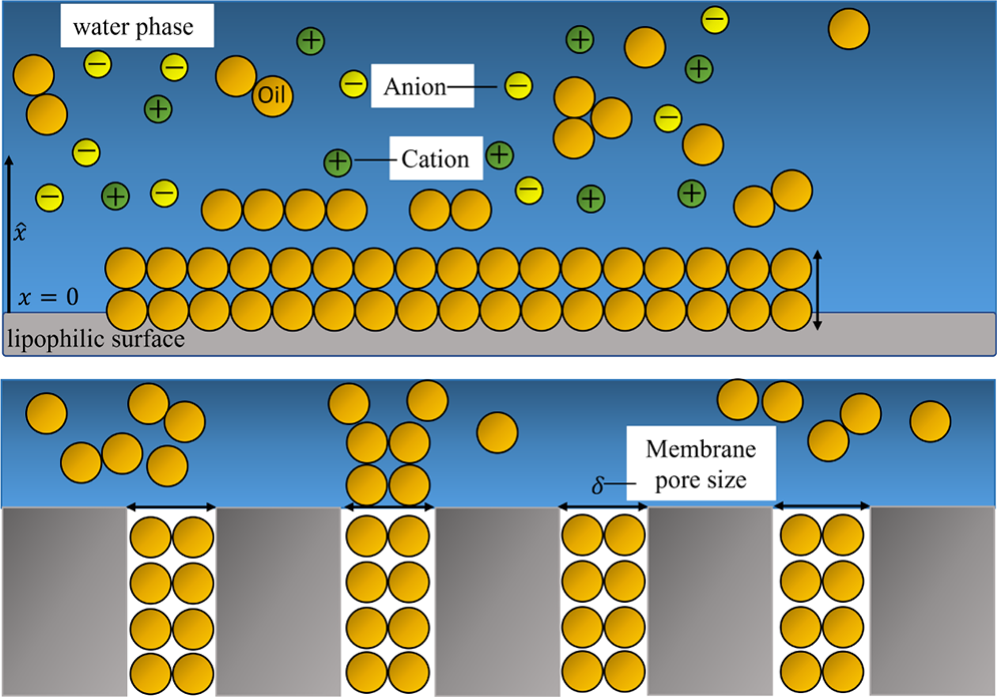
A semi-infinite system with a zoom-in of the lipophilic solid surface (in gray): The oil droplet spheres (in light brown) go toward the membrane due to their chemical affinity to the lipophilic surface. The continuous phase is water with anions and cations dispersed in it. δ is the pore size of the membrane.

The equilibrium density profile satisfies a vanishing variational derivative of the grand canonical ensemble , where the Lagrange multiplier, μ, is the chemical potential and is a constant. Further assuming that the density of the emulsion droplets changes solely normal to the solid surface of the membrane, along the coordinate *x* in Figure 4, we write the result as the simple unidirectional and dimensionless boundary value problem,
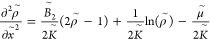
2where
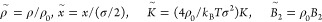
and . We solved the problem using the finite difference including the Simpson method for integration and the implicit matrix method for solving (eq 2). We required relative solution precision of 10^–4^ using norm two and norm infinity of oil droplet concentration distribution.

**Figure 5:**
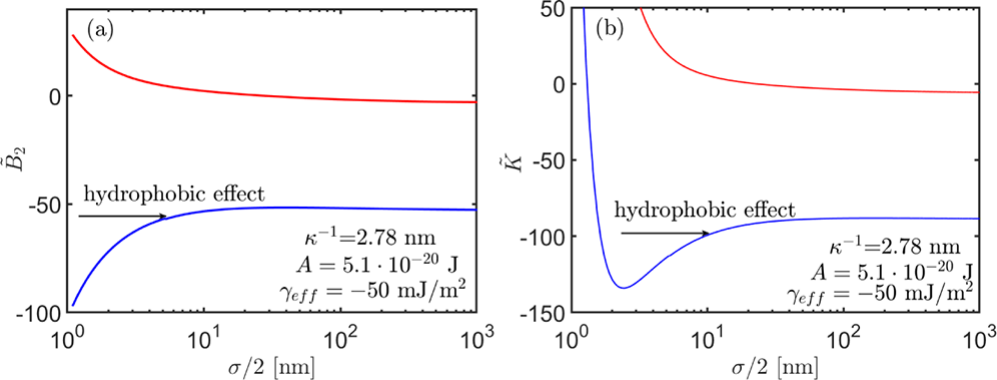
(a) Oil droplet size variations of the coefficients  and (b) , in the absence (red) and presence (blue) of hydrophobic forces.

In Figure 5, we present variations of the coefficients  and  for different oil radii in the range,  . Both coefficients decrease monotonically when the droplet size is increased (red curves). This is a manifestation of the increase in contributions from mean field colloidal forces relative to particle finite volume effects as the particle size decreases. The balance between contributions to the interaction potential from the finite volume of particles and mean field colloidal forces results in a change in the sign of the coefficient *B*_2_ when changing the radius of droplets, . Contributions from the attractive hydrophobic forces to the coefficients render  and  negative within the particle radii range (blue curves).

The chemical potential, μ, is to be determined during the solution of the problem. We set the characteristic density of droplets to be , which corresponds to a pure phase of the liquid in the droplet. A strong affinity between a membrane surface and the droplets usually corresponds to coating the membrane by a material of a similar chemistry to the droplets. This is represented by assuming the boundary condition  at the membrane surface. Far from the membrane, we assume that the concentration of the emulsion is the bulk concentration by requiring  and .

Focusing on the oil droplets within a water based electrolyte solution, we analyze the contribution of attractive and repulsive forces between oil drops to the accumulation of droplets next to the membrane surface. The excess concentration of droplets next to the membrane indicates the efficiency of membrane separation through the pores. Larger excess concentration of droplets next to the membrane surface corresponds to a larger excess concentration of droplets in membrane pores, see Figure 4.

**Figure 6:**
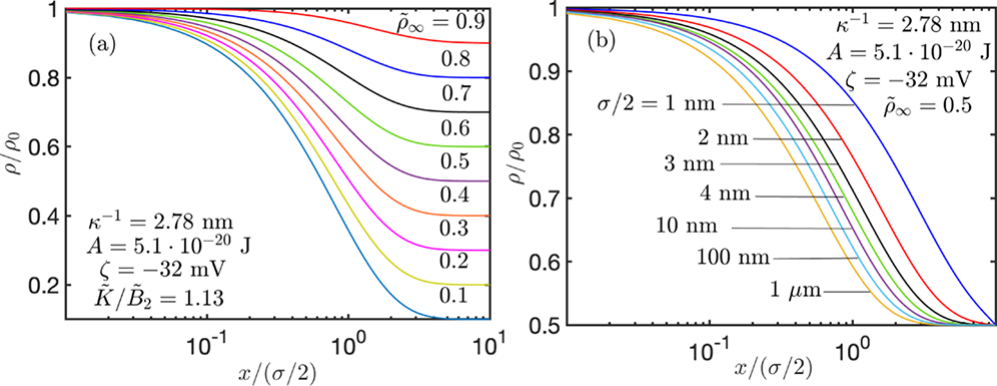
Dimensionless spatial variations of oil droplet number density under vdW and EDL interactions for (a) different concentrations at the bulk of liquid () and (b) different particle radii ().

In Figure 6a, we show the oil droplet density, ρ, as a function of separation from the membrane surface, *x*, for different bulk drop densities (emulsion concentration in the bulk of liquid), . Variations in ρ commence at ρ_0_ at the membrane surface, which corresponds to a membrane surface made from a similar chemistry to that of the oil. Greater dilution in the bulk concentration  renders greater variations in ρ, albeit, within the limits of our parametric study, the thickness of the high oil concentration layer near the membrane surface is approximately the same thickness irrespective to the bulk oil concentration.

In Figure 6b, we further manifest the contribution of colloidal forces to the thickness of the excess oil layer near the membrane surface for droplets smaller than 1 μm. At greater droplet sizes, colloidal forces are shadowed by contributions from the finite volume of the droplets; the droplet concentration distribution curves merge with those of the 1 μm droplets in the figure. This finding is a product of the repulsive forces between droplets effectively increasing the volume of the drop and the attractive forces effectively decreasing the size of the drops under the hard sphere, and no drop coalescence assumptions are taken here. The long-range repulsive EDL force is characterized by a Debye length of approximately 2.8 nm. This is the effective increase in the drop size to leading order; hence, it gives small contribution to the drop concentration when the size of the latter is greater than 10 nm.

We assess the efficiency of oil separation by introducing the measure,
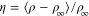
3where  is an averaging operator over the volume of the pores in the membrane. The measure captures the relative enhancement of oil concentration in the pores compared to that in the bulk mixture. It provides a measure for the membrane’s ability to possess excess oil concentration in its pore, which facilitates oil/water separation. To estimate the measure in (eq 3), we use a weak overlap approximation (an approximation commonly employed for approximation of EDL interactions.^32^ In this approximation, we employ the oil concentration  in our calculations depict in Figure 6 for a Cartesian geometry of a flat surface under a half space of oil/water mixture. The approximation is exact for pores that are much larger than the size of the oil drop and the characteristic decay length of the excess oil concentration away from the pore wall,^36^ so that the excess oil concentration near opposite sides of the wall weakly overlap and the curvature of the pore surface introduces a negligible contribution to the oil droplet concentration distribution. We thus approximate that , where δ is a representative pore diameter in the membrane and the factor of 2 accounts for the contribution to the excess oil concentration from the opposite side of a pore surface. We use this approximation to give an insight into membrane efficiency for our case study, where we estimate  for different oil drop sizes and representative membrane pore radii.

**Figure 7:**
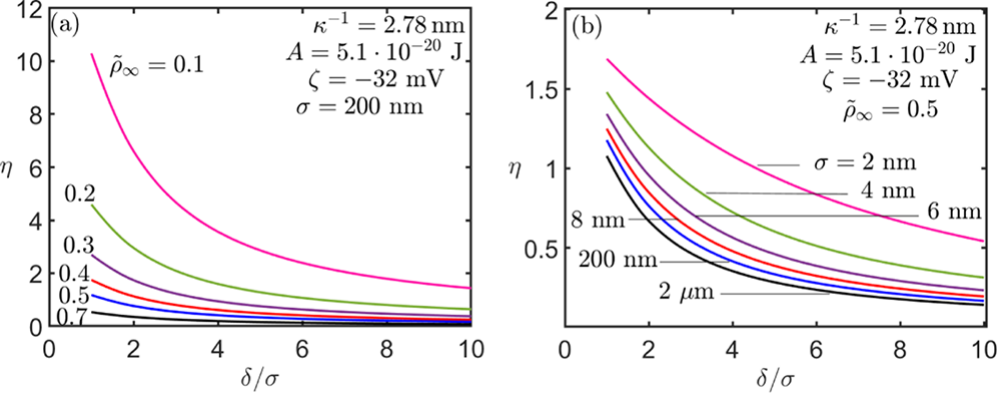
Membrane separation efficiency η as a function of membrane pore diameter δ for different values of: (a)  and (b) droplet diameter σ. The parameters used are ,  and .

Figure 7a depicts out insights into the membrane oil/water separation efficiency η as a function of the membrane dimensionless pore diameter , for bulk concentration , where σ is the oil droplet radii. We show that the oil/water separation efficiency decreases with the increase in the oil concentration in the bulk, . Moreover, as shown in Figure 7b, we examine the contribution of the oil drop size to the oil/water separation efficiency and find that smaller droplets render greater separation efficiency.

## Summary and Conclusions

Recently, the promise of using membranes to separate emulsions into their constituent phases has inspired an ever growing research effort. We contribute to this effort by considering a simple theory, considering colloidal forces and emulsion droplet concentration and size, yet ignoring, for simplicity, drop coalescence and deviations from sphericity to further connect the efficiency of a membrane for oil/water separation to various physical parameters. In our experiment-inspired analysis, we consider a case study of a hydrophobic membrane that supports the generation of oil-enriched emulsion in the pores therein, close to the membrane solid surface, and as a result in the emulsion past the membrane.

We use a case study to explore the connection between the efficiency of a membrane subject to its pore size and the colloidal forces and droplet size distribution in an oil-in-water emulsion for optimizing membrane properties for enriching the oil phase in the emulsion past the membrane and a depleted oil phase in the emulsion on the other side of the membrane as a result of mass conservation. In particular, we connect the emulsion droplet size and concentration and colloidal forces to the excess oil concentration near a hydrophobic membrane surface of affinity to the oil phase. For simplicity, we study the excess equilibrium concentration of oil droplets near a flat hydrophobic surface and consider the implications for the equilibrium concentration in membrane pores and thus to the membrane efficiency.

In our case study, we observe that contributions of colloidal forces to the excess oil layer thickness near the membrane surface become appreciable for droplets that are smaller than 1 μm. This is expected since colloidal (surface) forces dominate over other contributions to emulsion stability and therefore droplet distribution at particle sizes below 1 μm. Moreover, while our analysis is relevant to the physical parameters given in our case study, the general insight that colloidal forces will contribute to the thickness of the excess concentration of oil droplets near the membrane surface is generic. Assuming spherical and noncoalescing emulsion droplets (a kinetically stable emulsion), treating the emulsion using ideas from the theory for a nonideal gas gives that repulsive colloidal forces, e.g., the EDL force, increase the *effective* size of the emulsion droplets during interactions. Attractive colloidal forces, e.g., van der Waals (vdW) and hydrophobic forces, decrease the effective size of the emulsion droplets. Hence, in the absence of drop coalescence, colloidal forces effectively increase or decrease the effective size of the droplets, which alters the internal structure of the emulsion near the membrane surface.

In our case study, the repulsive EDL force is the longest range droplet–droplet interaction force and satisfies a Debye screening length of just under 3 nm. Hence, we observe an appreciable contribution to the increase in the emulsion concentration near the membrane surface for droplets whose radii are smaller than 10 nm. Colloidal interactions add small contributions to the concentration distribution of larger droplets whose radii are smaller than the Debye screening length. Moreover, we observe an increase in the excess oil concentration in a layer of approximately 10 drop radii thickness near the membrane surface for drops smaller than 10 nm. In the case of larger drops, we observe an excess oil concentration in a layer thickness of approximately 2 drop radii. These values depend on the physical parameters in our case study. Membrane pores with a diameter that corresponds to these thicknesses will support an increased oil concentration (oil enriched emulsion) in the pores, relative to the one in the bulk of the emulsion. Using our case study, we show that the distribution of oil concentration next to the hydrophobic membrane surface determines the oil/water separation efficiency according to the oil concentration in the bulk emulsion, the size of the droplets, the colloidal forces in the emulsion, and the size of the pores in the membrane.

## Supporting Information Available

The mathematical analysis of the correction to the vdW interactions and aspects of the numerical analysis used (PDF)
