# Supplementary material for: Contributions of Colloidal Forces to the Heterogeneous Separation of Stable Oil-In-Water Emulsions
Source: Langmuir. 2024 Oct 28;40(44):23458–64. doi: 10.1021/acs.langmuir.4c03056 (PMC11542182; doi:10.1021/acs.langmuir.4c03056)
Supplement: Supplementary file 1 — la4c03056_si_001.pdf [file la4c03056_si_001.pdf]

Supporting Information

Contributions of Colloidal Forces to the Heterogeneous Separation of Stable Oil-in-Water Emulsions

Roi Bar-On[†] and Ofer Manor[*,‡]

[†]*Applied mathematics department, Technion - Israel Institute of Technology, Haifa, 3200000, Israel. Currently at Institut de Biologie de l'École Normale Supérieure ENS, Paris*

[‡]*Department of Chemical Engineering, Technion - Israel Institute of Technology, Haifa, 3200000, Israel*

E-mail: manoro@technion.ac.il

Number of pages: 3

Number of figures: 0

Number of schemes: 0

Number of tables: 0

Table of Contents

vdW Interactions Correction **S2**

Numerical analysis of the problem and comparison to the analytical result **S3**

vdW interactions correction

The interaction energy between colloidal particles W is given as a summation over the pair-wise interaction energy between molecular segments, $U = -C/r^6$, as

$$W = \frac{k_B T}{2} \int \int \rho(r)\rho(r') \left(1 - e^{-U/k_B T}\right) dr dr'. \tag{S1}$$

We expand the exponent in small U, $e^{-U/k_B T} = 1 - U/k_B T + U^2/2(k_B T)^2 + \ldots$, and substitute the result in (S1) to obtain that

$$W = \frac{1}{2} \int \int \rho(r)\rho(r') \left(\frac{-C}{r^6} + \frac{C^2}{2r^{12}} \frac{1}{k_B T} + \ldots\right) dr dr'. \tag{S2}$$

The first term in the brackets on the right hand side of the equation is a textbook problem and translates for to the usual Van der Waals interaction energy between objects when ignoring the interaction of molecular segments within the same object. For example, the interaction energy per unit area between two parallel infinite slabs, which are comprised from molecular segments of constant density ρ_{ms}, is $W_{\text{flat}}^{(1)} = -A/12\pi l^2$, where the slabs are separated by a distance l and $A \equiv \pi^2 C \rho_{ms}^2$. The second term in the brackets gives the correction to the interaction energy per unit area,

$$W_{\text{flat}}^{(2)} = \frac{A^2}{6! k_B T \rho^2 \pi^3 l^8}, \tag{S3}$$

under the same assumptions. The correction to the interaction energy becomes appreciable at small separations between the particles, i.e., at separations of approximately 1 nm and less – see figure 2(a) for example. The Derjaguin approximation translates the expression in (S3) to the interaction energy between two spherical particles of radius R,

$$W_{VdW,rep}(z) = -\int_{l \to \infty}^{l = z - 2R} \pi R W_{\text{flat}}^{(2)} dl = \frac{A^2 R}{7! k_B T \rho_{ms}^2 \pi^2 (z - 2R)^7} = W''_{VdW}, \tag{S4}$$

which we use in the main text.

Numerical analysis of the problem and comparison to the analytical result

Denoting the different parameters in (6) as $\bar{\mu} = \frac{\tilde{\mu}}{\tilde{K}}, b = -\frac{\tilde{B}_2}{\tilde{K}}, a = \frac{1}{\tilde{K}}$, $y = \rho$ and $x = \tilde{x}$ gives the following equation to be solved:

$$y'' + aln(1-y) + by + \bar{\mu} = 0. \tag{S5}$$

Employing central difference approximation for the derivative we obtain that

$$y_{i+1} - 2y_i + y_{i-1} + a\Delta\tilde{x}^2 ln(1-y_i) + b\Delta\tilde{x}^2 y_i + \Delta\tilde{x}^2\bar{\mu} = 0, \tag{S6}$$

and the boundary conditions are translated to $y_0 = \rho_0,\ y_\infty = \rho_\infty$. Moreover, the chemical potential is evaluated far from the solid surface, using the requirement that the concentration should be independent of the spatial coordinate in the bulk of the solution,

$$\bar{\mu} = -aln(1-y_\infty) - by_\infty. \tag{S7}$$

The problem translates to the matrix form:

$$
\begin{pmatrix}
\alpha-2 & 1 & 0 & . & . & . & . & . & . & 0 \\
1 & \alpha-2 & 1 & 0 & . & . & . & . & . & 0 \\
0 & 1 & \alpha-2 & 1 & 0 & . & . & . & . & . \\
. & . & . & . & . & . & . & . & . & . \\
. & . & . & . & . & . & . & . & . & . \\
. & . & . & . & . & . & . & . & . & . \\
. & . & . & . & . & . & . & . & . & . \\
. & . & . & . & . & . & . & . & . & . \\
. & . & . & . & . & . & . & 1 & \alpha-2 & 1 \\
. & . & . & . & . & . & . & . & 1 & \alpha-2
\end{pmatrix}
\begin{pmatrix}
y_1 \\ . \\ . \\ . \\ . \\ . \\ . \\ . \\ . \\ y_{N-1}
\end{pmatrix}^{(k+1)}
=
\begin{pmatrix}
\beta_M ln\left(\frac{1-y_\infty}{1-y_1}\right)+\alpha y_\infty - y_0 \\
\beta_M ln\left(\frac{1-y_\infty}{1-y_2}\right)+\alpha y_\infty \\
\beta_M ln\left(\frac{1-y_\infty}{1-y_3}\right)+\alpha y_\infty \\
. \\ . \\ . \\ . \\ . \\ . \\
\beta_M ln\left(\frac{1-y_\infty}{1-y_{N-1}}\right)+(\alpha-1)\, y_\infty
\end{pmatrix}^{(k)},
\tag{S8}
$$

which we solve by iteration, where $\alpha = b\Delta\tilde{x}^2, \beta_M = a\Delta\tilde{x}^2$, with the unknown $\bar{\mu}$, and where k is a running index symbolizing the number of iteration.
